# Smartphone-Based Virtual and Augmented Reality Implicit Association Training (VARIAT) for Reducing Implicit Biases Toward Patients Among Health Care Providers: App Development and Pilot Testing

**DOI:** 10.2196/51310

**Published:** 2024-03-07

**Authors:** Jiabin Shen, Alex J Clinton, Jeffrey Penka, Megan E Gregory, Lindsey Sova, Sheryl Pfeil, Jeremy Patterson, Tensing Maa

**Affiliations:** 1 Department of Psychology, University of Massachusetts Lowell, Lowell, MA, United States; 2 LittleSeed, Inc, Columbus, OH, United States; 3 Department of Health Outcomes & Biomedical Informatics, College of Medicine, University of Florida, Gainesville, FL, United States; 4 Center for Advancement of Team Science, Analytics, and Systems Thinking in Health Services and Implementation Science Research, College of Medicine, Ohio State University, Columbus, OH, United States; 5 College of Medicine, Ohio State University, Columbus, OH, United States; 6 Advanced Computing Center for Arts and Design, Ohio State University, Columbus, OH, United States; 7 Center for Clinical Excellence, Nationwide Children’s Hospital, Columbus, OH, United States

**Keywords:** implicit bias, health care, Medicaid, virtual reality, augmented reality, smartphone, mHealth, mobile app, innovative, implicit bias training program, sexual orientation, sexual orientations, gender identity, gender identities, gender preferences, gender preference, efficacy, health care providers, health care provider, socioeconomic, mobile application, training, XR, extended reality

## Abstract

**Background:**

Implicit bias is as prevalent among health care professionals as among the wider population and is significantly associated with lower health care quality.

**Objective:**

The study goal was to develop and evaluate the preliminary efficacy of an innovative mobile app, VARIAT (Virtual and Augmented Reality Implicit Association Training), to reduce implicit biases among Medicaid providers.

**Methods:**

An interdisciplinary team developed 2 interactive case-based training modules for Medicaid providers focused on implicit bias related to race and socioeconomic status (SES) and sexual orientation and gender identity (SOGI), respectively. The simulations combine experiential learning, facilitated debriefing, and game-based educational strategies. Medicaid providers (n=18) participated in this pilot study. Outcomes were measured on 3 domains: training reactions, affective knowledge, and skill-based knowledge related to implicit biases in race/SES or SOGI.

**Results:**

Participants reported high relevance of training to their job for both the race/SES module (mean score 4.75, SD 0.45) and SOGI module (mean score 4.67, SD 0.50). Significant improvement in skill-based knowledge for minimizing health disparities for lesbian, gay, bisexual, transgender, and queer patients was found after training (Cohen *d*=0.72; 95% CI −1.38 to −0.04).

**Conclusions:**

This study developed an innovative smartphone-based implicit bias training program for Medicaid providers and conducted a pilot evaluation on the user experience and preliminary efficacy. Preliminary evidence showed positive satisfaction and preliminary efficacy of the intervention.

## Introduction

### Implicit Bias in Health Care Settings

Defined as unconscious associations or negative evaluations of a person or group of people on the basis of nonrelevant characteristics [1], implicit biases have been found to be prevalent among the general population against “marginalized’ groups such as those from minority racial, ethnic, or socioeconomic backgrounds [2]. Implicit biases, which present in health care settings as irrational and unconscious perceptions, stereotypes, or prejudices among health care providers when interacting with patients, are especially concerning [3]. Past research has found that implicit bias in health care settings is associated with a decrease in overall quality of care, with impacts including increased risk of misdiagnosis [4-7], inaccurate patient pain perception [8,9], differential treatment recommendations for patients who belong to sexual orientation or gender identity minority groups [10-12], and negative perceptions of patients from racial minority backgrounds [13-17]. Implicit biases may also exist during interactions between health care professionals, such as selection bias when choosing candidates for future health care practitioner residency [18], which may have wider implications for the quality and safety of patient care. Furthermore, such implicit biases have been found within adult and pediatric health care settings [19] across medical conditions including ADHD, asthma, cardiology, and child abuse, which could affect quality of care for these vulnerable populations [20-22].

### Existing Efforts to Reduce Implicit Bias in Health Care Settings

In response, increasing efforts have been devoted to addressing the significant threat posed by implicit bias toward health care services and patient outcomes. The first type of interventional efforts focus on “environmental engineering,” with the goal to minimize mechanisms in health care settings that may give rise to biased interactions between health care professionals and patients. One example of this type of intervention is the development and implementation of automatic patient care prompts through electronic portals, where computer algorithms are standardized for all patients regardless of sociodemographic backgrounds, attempting to reduce opportunities for human interference (beyond the algorithm development phase) [23]. A second type of intervention uses cognitive rehearsal to walk practitioners through potentially harmful scenarios to practice their ideal response; this has shown promise at changing health care practitioner behavior to reduce bullying and workplace turnover [24,25]. While not widely used in combatting health care bias explicitly, the methodology shows a clear avenue for its application to bias training.

A third type of intervention, which will also be the focus of this study, attempts to develop educational programs with the goal of improving knowledge and awareness of implicit bias among medical students or health care professionals, which can range from traditional educational seminars to experience-oriented storytelling, to highlight the importance of patient perspectives in daily practice [26-28]. Such efforts have so far yielded positive results where health care professionals were found to become more aware of their own biases and have resulted in improved communication between health care professionals and marginalized patient groups [29,30].

### Application of Augmented Reality–Based Medical Training

Despite the promising results from educational programs in the existing literature, one limitation in existing approaches for implicit bias training is the lack of immersive learning experiences that may provide optimal learning outcomes and behavior changes. As a cutting-edge technology that prioritizes experiential learning, virtual reality (VR) and augmented reality (AR) could provide an ideal solution with immersive learning experiences for implicit bias training. For example, one recent study examined biases during interactions between virtual health care providers and virtual patients for medical triage training. Regardless of the skin tone of the avatar (ie, the health care provider), it took participants more time to initiate assistance and they were more likely to make errors when triaging dark-skinned virtual patients compared to light-skinned virtual patients [31].

AR, as a more recent member of the x-reality technologies, is posed to offer an even better learning experience that combines the immersion provided by VR and tailored customization that adapts to users’ dynamic environments. Adoption of AR in medical education has been found in a wide range of medical branches from surgery (eg, laparoscopic procedure training) to anatomy [32]. Furthermore, because AR-based training is readily available on consumer-grade mobile devices such as smartphones and tablets, its mobility provides medical professionals with remote accessibility to training content regardless of their physical location (this advantage has been further acknowledged during the COVID-19 pandemic) [33]. However, despite the increasing adoption of AR in medical training, a recent systematic review has found little evidence on the availability of AR-based implicit bias training among health care professionals in the literature [33].

### This Study

To address this important gap, this study aimed to develop a mobile training program, VARIAT (Virtual and Augmented Reality Implicit Association Training), specifically for improving the awareness of implicit biases among health care providers when interacting with patients in daily practice. The design considerations for developing this novel AR-based implicit bias training program are described, followed by a preliminary examination of initial user feasibility and learning outcomes, including user reactions; relevance to practice; and changes in knowledge, attitudes, and behavioral skills related to implicit bias before and after receiving the training program.

## Methods

### Designing the VARIAT Program

#### Overview of Technical Design Considerations

The VARIAT program focused on delivering an immersive, interactive learning experience to the broadest possible audience in self-contained segments, allowing users to complete the training over time and a variety of sessions while retaining their progress across sessions. When building the 3D worlds for delivery on the broadest number and sizes of mobile devices, simplified, realistic, and familiar spaces were built, including offices, lobbies, and examination rooms where the learner could experience the simulations. Characters in the world were designed with exaggerated cartoon features to provide visual distinction with skin tone, hair, size, outfits, and accessories, while minimizing unnecessary details and maximizing ease of recognition for interaction on mobile-sized screens. The approach to world design addressed design and performance considerations, allowing production of additional characters and scenarios without significant technical overhead in either the creation process or the learner’s experience on their device. The dialogue and training content was presented via text.

#### Hardware Requirements and Considerations

One key goal of the VARIAT program was the need to maximize audience reach and minimize specialty equipment for learners to access the content. At the time of its development, most iPhones and Android devices had the cameras, accelerometers, and gyroscopes needed to provide users the ability to see into and navigate virtual worlds by simply holding up and moving their devices. When the VARIAT program was introduced, learners needed at least an iPhone 8 with iOS 13 or an Android device running Android 9 or higher.

#### Software Requirements and Considerations

The maturity of the mobile app environment offers many development tools and approaches for developing mobile apps. For the VARIAT program, the developers used the Unity game engine (Unity Technologies) for game content with ARKit (Apple Inc) and ARCore (Google LLC) for the augmented reality component and deployed both iOS and Android apps that were readily available in their respective app stores. Blender (Blender Foundation) was used for 3D modeling and animation, and Photoshop (Adobe) was used to create 2D assets.

The learner downloads the app from the Apple App Store or Google Play on their device, and their progress is maintained on the device with evaluation, progress, and study data synchronized as the learner completes various modules. When synchronized, the data are stored and managed using Google Big Query Workspace, which produces data feeds for training evaluators and researchers.

### Overview of Content Design Considerations

#### Training Framework of the VARIAT App

The VARIAT game was designed based on the integration of evidence-based cognitive psychology with the latest simulation technologies, including VR (eg, a simulated experience of interacting with a virtual clinician-patient scenario that is vulnerable to implicit biases in a virtual environment using 6-degrees-of-freedom motion- and gesture-based interactions) and AR (eg, converting a user’s physical environment into a clinic’s waiting room for interactive experiences). The goal of the app is to improve awareness of implicit biases among Medicaid providers, to educate them on how these biases can lead to inequitable care, and to offer strategies and resources that may minimize health disparities. This mobile app can be installed on any Apple iOS and Android device and is designed to be completed in one sitting or in short segments.

The game consists of 2 distinct but interconnected modules, targeting implicit biases within medical settings toward patients from minority racial backgrounds, with low socioeconomic status (SES), or from a minority sexual orientation or gender identity (SOGI) group. Learners enter an AR-based interactive role-playing game, in which they encounter a series of 6 scenarios. Each scenario takes approximately 5 minutes to complete and is related to the specific implicit bias being addressed in that particular module (Race/SES or SOGI). Each scenario within the module is designed to address specific issues related to disparities in medical settings that relate to the overall theme of the module. The primary outcome measure of this training program is to evaluate providers’ attitudes and beliefs on key concepts related to implicit biases and health disparities in a medical setting before and immediately following the training.

#### Race/SES Module

The first module within the VARIAT mobile game is the race/SES module, which consists of 3 scenarios dealing with issues of racial bias, transportation and food instability, and implicit bias. The first scenario in this module addresses issues of racial bias within a health care setting and prompts the user to consider how issues of racial and ethnic identity could impact treatment recommendations and the resulting care for patients of minority groups. The next scenario is designed to promote self-reflection on how socioeconomic factors like unreliable transportation or housing could affect a patient’s ability to show up for health care visits or comply with care recommendations by medical providers. The last scenario is designed to help users understand how implicit bias from medical providers could impact patient perception and negatively impact patient care.

Each scenario contains prompts where the user is asked to make a choice about the “case” presented within the VARIAT AR game. The user is then given information about the scenario and resources for how to better understand the specific issues for each scenario with the goal to educate them on how to improve practitioner behavior as it relates to the theme of the module. A summary of the scenarios and objectives for this module can be found in Multimedia Appendix 1.

#### SOGI Module

The second module in the VARIAT mobile game is the SOGI module, which consists of 3 scenarios dealing with issues of SOGI implicit bias, inclusivity for patient care settings, and lesbian, gay, bisexual, transgender, queer (LGBTQ+) patient considerations. The first scenario helps portray the way that microaggressions and implicit biases in patient-provider communication can promote negative disparities in treatment for SOGI minority patients. The second scenario asks users to design their own patient waiting room and helps educate and guide users on what considerations should be made to ensure a medical setting is a safe and welcoming environment for SOGI minority patients. The last scenario in the module helps users recognize the harmful effects of biased behavior toward LGBTQ+ patients and offers space for self-reflection on how to reduce enacted bias for this patient group.

The scenarios in this module also contain prompts for users to answer to better assess their understanding of key concepts or takeaways from each scenario. The SOGI module places an increased emphasis on self-reflection as the scenarios are designed to help users draw parallels to their own experiences in medical practice through reflective exercises following the conclusion of each scenario within the module. A summary of the scenarios and objectives for this module can be seen in Multimedia Appendix 2.

#### User Workflow

On start-up, users are given some brief instructions on how to prepare themselves for immersion in the VARIAT AR game. Users are then instructed to select any available module to start engaging with the content within. Once a module is selected, participants are placed in a virtual hospital setting and can check on the various patients within. When selected in the AR game, these patients display information on their illnesses and present the user with additional narratives about the patients from the “staff” in the AR game. Users are then given different decision options on what to do for each patient’s individual case. After helping these patients, the user is provided with information and resources that relate to the content of the module. Unbiased choices “score” higher than choices that are considered to have been influenced by implicit biases toward marginalized patients. After completing the tasks in their module, the users are given a summary of their scores for that module with feedback on how to improve, and additional information to support that improvement relative to the context of their scenario. After completing a module, users are sent back to the home screen, where they can replay the same module or select a new module to explore. A depiction of the app layout, user experience, and scenario prompts is presented in Multimedia Appendix 3.

### User Experience and Preliminary Efficacy of the VARIAT Program

#### Participants and Procedure

Eighteen clinicians (n=12 female) who were predominantly White (non-Hispanic) participated in the VARIAT training. Physicians comprised 8 of the 18 (44%) participants, and 12 of the 18 participants had more than 5 years’ experience in health care. The most common workplace setting was hospitals, with private practices, health care system–affiliated clinics, and other workplace settings reported as well. Most participants estimated that Medicaid patients comprised more than 30% of their total caseload, with reported ages of patients seen varying between children, adults, and older adults. Demographic information is reported in Table 1 for the total number of participants (N=18), participants who participated only in the race/SES module (n=7), participants who participated in only the SOGI module (n=5), and participants who completed both the race/SES and SOGI modules (n=6). Participants were recruited through professional networks and were eligible for the study if they were Medicaid providers.

**Table 1.** Demographic information for the participants.

Variables		Overall (n=18), n (%)	Race/SES^a^ only (n=7), n (%)	SOGI^b^ only (n=5), n (%)	Race/SES and SOGI (n=6), n (%)
**Race/ethnicity**					
	White (non-Hispanic)	16 (88)	6 (85)	5 (100)	5 (83)
	Non–White^c^	2 (11)	1 (14)	0 (0)	1 (16)
**Gender**					
	Male	6 (33)	2 (28)	2 (40)	2 (33)
	Female	12 (66)	5 (71)	3 (60)	4 (66)
	Prefer not to say	0 (0)	0 (0)	0 (0)	0 (0)
**Profession**					
	Medical resident	3 (16)	2 (28)	0 (0)	1 (16)
	Nurse	0 (0)	0 (0)	0 (0)	0 (0)
	Fully credentialed physician	8 (44)	3 (42)	2 (40)	3 (50)
	Social worker	5 (27)	1 (14)	2 (40)	2 (33)
	Other	2 (11)	1 (14)	1 (20)	0 (0)
**Work setting**					
	Health care system–affiliated clinic	2 (11)	1 (14)	1 (20)	0 (0)
	Hospital	9 (50)	3 (42)	3 (60)	3 (50)
	Private practice	2 (11)	1 (14)	0 (0)	1 (16)
	Other	4 (22)	2 (28)	1 (20)	1 (16)
	Missing	1 (5)	0 (0)	0 (0)	1 (16)
**Experience in work setting**					
	Less than 1 year	0 (0)	0 (0)	0 (0)	0 (0)
	1-5 years	6 (33)	3 (42)	1 (20)	2 (33)
	6-10 years	3 (16)	2 (28)	1 (20)	0 (0)
	11-15 years	2 (11)	1 (14)	0 (0)	1 (16)
	16-20 years	0 (0)	0 (0)	0 (0)	0 (0)
	21-25 years	2 (11)	1 (14)	1 (20)	0 (0)
	26-30 years	2 (11)	0 (0)	1 (20)	1 (16)
	31 years or more	2 (11)	0 (0)	1 (20)	1 (16)
	Missing	1 (5)	0 (0)	0 (0)	1 (16)
**Percentage of Medicaid patients seen**					
	Less than or equal to 30%	4 (22)	2 (28)	1 (20)	1 (16)
	Greater than 30%	12 (66)	5 (71)	4 (80)	3 (50)
	I do not see Medicaid patients	1 (5)	0 (0)	0 (0)	1 (16)
	Missing	1 (5)	0 (0)	0 (0)	1 (16)
**Age of patients^d^**					
	Children	10 (55)	5 (71)	3 (60)	2 (33)
	Adults	11 (61)	0 (0)	2 (40)	2 (33)
	Older adults	8 (44)	5 (71)	2 (40)	1 (16)
	I do not see Medicaid patients	1 (5)	0 (0)	0 (0)	1 (16)

a SES: socioeconomic status.

b SOGI: sexual orientation and gender identity.

c Combined category.

d Multiple answers selected.

Questionnaires were administered to participants remotely through the VARIAT app, and data collection took place from March to June 2020.

### Measures

#### User Experience Measures

Users’ reactions to both the race/SES and SOGI modules of the VARIAT program were assessed by asking participants about their perception of the modules after the test. After experiencing the AR simulation, users were asked questions designed to test their engagement with the AR experience, such as if they felt a sense of “being there” in the AR experience or how real they found the AR experience to be. These answers were scored on a scale of 1 to 5, with higher scores indicating stronger agreement. Participants were also asked questions about how they might apply the AR experience to their job with questions such as “How do you think this training will help you on the job (Mark all that apply)?” with different response items to assess perceived benefits from the training. These items were scored using dichotomous coding for each option (0 for not applicable and 1 for applicable).

#### Preliminary Efficacy Measures

Training outcomes were reported through changes in affective knowledge and changes in skill-based knowledge measured by comparing pre-post test responses. Affective knowledge (items assessing how participants expect their perceptions to impact their patients) was measured by agreement with items that were adapted from the California Brief Multicultural Competence Scale [31]. Example items include the following: “I am aware of how my own values might affect my patients” or “I am aware of institutional barriers that affect patients.” Skill-based knowledge was assessed differently for the race/SES and SOGI modules, with questions referring to each respective population focused on in the module. Race/SES skill-based knowledge was measured by rating participant agreement with the following internally developed statements: “I am confident that I can recognize the role that implicit bias plays in leading to inequitable care for patients of low socioeconomic status,” and “I am confident that I can apply strategies and use resources to minimize health care disparities for patients with low socioeconomic status.” SOGI skill-based knowledge was measured similarly, with “race/SES population” being replaced with “LGBTQ+ population” in the skills-based questions. Training outcomes were reported for each module separately, with the race/SES module (n=13) and SOGI module (n*=*11) consisting of all participants that completed each module. All measures were scored on a scale of 1 to 5, with higher scores representing stronger agreement.

#### Data Analysis Plan

All analyses were conducted using SPSS Statistics (version 27.0; IBM Corp). Demographic characteristics were described using frequencies and percentages for the categorical variables. Demographic characteristics were reported across 4 participant groupings: participants who took only the Race/SES module, participants who took only the SOGI module, participants who took both the Race/SES and SOGI modules, and an overall group of all unique participants.

After testing for normality using the Shapiro-Wilk test, the training reactions and pre-post skills and attitude outcome data were found to not be normally distributed (*P*<.001). As a result, we used nonparametric tests for analyzing these 2 outcome domains. For the usability data, we used the Wilcoxon signed-rank test to measure the continuous reaction items and report the mean and SD for participants who used both the race/SES module and the SOGI module. The categorical training reaction items were reported using frequencies and percentages. For analyzing the skills and attitudes outcome data, the Wilcoxon matched-pairs signed-rank test was used to report the mean, SD, effect size (Cohen *d*), and 95% CI for pre-post changes in scores. The scores for each module were analyzed separately for all participants who took each respective module. Given that some participants completed both modules (n*=*6), there is a small amount of overlap in participant representation across all reported outcome data. All data and study materials will be made available on request.

### Ethical Considerations

The Ohio State University (OSU) Institutional Review Board has determined this study was exempt from review according to the Policy on Human Subjects Research of the OSU Human Research Protection Program.

## Results

### User Experience (Training Reactions)

For perception of the AR experience, participants who received training in the race/SES and SOGI modules reported similar ratings for the overall AR experience. Participants reported positive feelings of “being there” (race/SES module: mean score 4.62, SD 1.56; SOGI module: mean score 3.91, SD 1.97) and high relevance of the AR training to their respective jobs (race/SES module: mean score 4.75, SD 0.45; SOGI module: mean score 4.67, SD 0.50) across both modules. Participants across both modules perceived the AR experience as being “a little” realistic, with the SOGI participants reporting less realism on average (mean score 2.91, SD 1.64) compared to the race/SES participants (mean score 3.77, SD 1.83). For the reported intention to apply the AR experience to their jobs, only the participants who received training in the race/SES module responded to this item. On average, these participants reported that they were less likely to apply the AR experience to their jobs (mean score 2.31, SD 1.11).

Assessing the perceived benefits of the AR experience to the participants’ jobs revealed that the race/SES and SOGI modules had some key differences in support across beliefs. Participants from both the race/SES and SOGI modules reported varying levels of positive agreement that the experience could improve their relationships with their patients (8/11, 73% SOGI participants; 8/13, 62% race/SES participants) and avoid undesirable events in patient care (8/11, 73% SOGI participants; 8/13, 62% SES participants). Conversely, 9 of 11 SOGI participants (82%) showed adequate agreement with the belief that the training would help improve tailored care and 7 of 11 participants (64%) believed that the training would improve patient satisfaction. For the race/SES participants, 7 of 13 (54%) showed moderate agreement with beliefs about improving tailored care, while 6 of 13 (46%) agreed that the training could improve patient satisfaction. The race/SES participants showed higher agreement with the belief that the module would improve their community resources (9/13, 69%) compared to the SOGI module participants (5/11, 45%). A detailed summary of user experience findings is reported in Table 2.

**Table 2.** User experience (training reaction) outcomes.

Variables		Race/SES^a^ (n=13)	SOGI^b^ (n=11)	Effect size (Cohen *d*)
**Augmented reality experience scores, mean (SD)**				
	Feeling of ”being there”^c^	4.6 (1.6)	3.9 (2.0)	0.40
	Realism of augmented reality^c^	3.7 (1.8)	2.9 (1.6)	0.49
	Relevance to job^d^	4.8 (0.5)	4.7 (0.5)	0.17
	Intention to apply augmented reality experience^d^	2.3 (1.1)	N/A^e^	N/A
**Participants reporting applicability to job, n (%)** ^f^				
	Improve relationship with patients	8 (62)	8 (73)	N/A
	Improve patient satisfaction	6 (46)	7 (64)	N/A
	Improve tailored care	7 (54)	9 (82)	N/A
	Avoid undesirable events	8 (62)	8 (73)	N/A
	Improve community resources	9 (69)	5 (45)	N/A
	Other benefit	0 (0)	1 (9)	N/A

a SES: socioeconomic status.

b SOGI: sexual orientation and gender identity.

c Measured on a scale from 1=not at all to 7=very much.

d Measured on a scale from 1=strongly disagree to 5=strongly agree.

e N/A: not applicable.

f Multiple answers selected.

### Preliminary Efficacy

For the skills questions, there was no significant difference in pre-post scores assessing the changes in awareness of implicit bias for patients of varying race/SES groups (pre: mean 4.31, SD 0.48; post: mean 4.46, SD 0.52; *d*=0.22; 95% CI −0.77 to 0.33) or the ability to manage health disparities caused by race/SES group (pre: mean 3.85, SD 0.56; post: mean 4.31, SD 0.48; *d*=0.52; 95% CI −1.10 to 0.07) . This pattern was true for measuring awareness of implicit bias for LGBTQ+ patients (pre: mean 4.36, SD 0.51; post: mean 4.73, SD 0.47; *d*=0.54; 95% CI −1.16 to 0.11). For minimizing health disparities realted to LGBTQ+ status, there was a significant difference between pre- and posttest scores (pre: mean 3.91, SD 0.94; post: mean 4.64, SD 0.51; *d*=0.72; 95% CI −1.38 to −0.04) with participants scoring closer to “strongly agree” after experiencing the AR experience.

For the attitudinal questions, there were nonsignificant improvements in the race/SES module in assessing how personal values affected patients (pre: mean 3.92, SD 0.95; post: mean 4.31, SD 0.84; *d*=0.44; 95% CI −1.01 to 0.14), how institutional barriers affect patients (pre: mean 4.23, SD 0.60; post: mean 4.31, SD 0.48; *d*=0.12; 95% CI −0.66 to 0.43), and participants’ ability to identify reactions based on stereotypes (pre: mean 4.15, SD 0.56; post: mean 4.38, SD 0.51; *d*=0.53; 95% CI −1.10 to 0.07). For the SOGI module, changes from pre to posttraining were also nonsignificant for all attitudinal items (pre: mean 4.18, SD 0.87; post: mean 4.45, SD 0.52; *d*=0.30; 95% CI −0.90 to 0.31), institutional barrier items (pre: mean 4.36, SD 0.67; post: mean 4.36, SD 0.67; *d*=0.00; 95% CI −0.59 to 0.59), and items related to identifying stereotypical reactions (pre: mean 4.36, SD 0.51; post: mean 4.36, SD 0.92; *d*=0.00; 95% CI −0.59 to 0.59). A summary of preliminary efficacy findings for each module can be found in Table 3.

**Table 3.** Race/socioeconomic status (SES) pre- and posttest skills and attitude outcomes (n=13).

Variables		Pretest score, mean (SD)	Posttest score, mean (SD)	Cohen *d (*95% CI)	
**Skills questions**					
	Implicit bias (race/SES)	4.3 (0.5)	4.5 (0.5)	0.22 (–0.77 to 0.33)	
	Minimize health disparities (race/SES)	3.9 (0.6)	4.3 (0.5)	0.52 (–1.10 to 0.07)	
**Attitudinal questions**					
	How my values affect patients	3.9 (1.0)	4.3 (0.5)	0.44 (–1.01 to 0.14)	
	How institutional barriers affect patients	4.2 (0.6)	4.3 (0.5)	0.12 (–0.66 to 0.43)	
	Identify reactions based on stereotypes	4.2 (0.6)	4.4 (0.5)	0.53 (–1.10 to 0.07)	

## Discussion

This study developed a VR and AR implicit association training program for Medicaid providers based on cognitive psychology and the latest mobile simulation technologies. Designed to improve awareness of implicit biases related to patients’ SES and sexual orientation/gender identity, learners are able to complete six 5-minute interactive role-playing scenarios on their smartphones. Results of pilot user experience research among 18 participants found adequate acceptability and preliminary efficacy (ie, a nonsignificant increase in most outcomes) of the VARIAT program. These findings are consistent with recent literature in cognitive psychology about the possible benefits of AR interventions for health care providers [34-37].

While researchers have spent the last 20 years attempting to reduce implicit bias [38-41], few attempts have been made to integrate the latest immersive technologies, such as AR and VR, with provider-level implicit bias training. For example, a recent meta-analysis of 492 interventions on implicit biases found only a handful of studies attempting to change implicit bias used any kind of VR or AR [42]. Narrowing down to implicit bias training in the health care setting, another recent literature review found few studies that focused on addressing bias at the provider level [43-46]. Therefore, while implicit bias in health care more broadly has been long recognized as a prominent issue [3], there is an important gap in research that develops technology-assisted training programs so that such programs can be more readily available for health care providers and so that implicit bias training can be received at a time and location that works best for them rather than having to attend in-person training sessions. The VARIAT program reported in this study addresses this critical literature gap by offering a convenient and publicly available program that can be integrated into medical training for health care professionals interacting with Medicaid patients, whose training may have important beneficial impacts on patients from disadvantaged backgrounds and those who experience reduced access to high quality of care due to multiple individual and societal barriers [47]. For example, the VARIAT program is brief and can be completed on a mobile device during “fragmented” time windows that fit within the often-chaotic work schedule of medical professionals. Therefore, medical institutions may consider integrating the VARIAT training as a regular refresh of lengthier and more comprehensive in-person or on-site bias-reduction training for their health care professional teams.

Furthermore, among the studies that focused on mitigating health care provider biases, few documented detailed feasibility and efficacy data [48-51]. This study is among the first in the literature to measure both positive provider reactions and efficacy outcomes at multiple levels, including user experience with AR, perceived utility in users’ professional work, and perceived attitudes toward patients and skills in mitigating implicit biases at work. It was interesting to find that although the study participants perceived relatively high levels of immersion (“being there”), AR realism, and job relevance from the VARIAT training, they expressed low levels of intention to apply this experience to their daily work. One possible explanation for this discrepancy might be the challenges of translating learned knowledge to behavioral changes, as commonly seen in educational interventions, potentially due to the limited scenarios provided by the training compared to the broad variations in participants’ own daily work experiences. The collection of both pre- and postintervention efficacy outcomes further allowed us to measure the potential interventional effects of each of the VARIAT training modules (race/SES and SOGI). However, it should be noted that this paper focused primarily on sharing with the scientific community the development processes and design considerations of a novel implicit bias training program for Medicaid providers. Therefore, caution should be applied when interpreting the preliminary results of this pilot user experience study.

### Study Limitations

There are several important limitations to this study. First, the current iteration of the VARIAT program is being delivered on mobile devices. This training program might elicit different user experiences and efficacy outcomes should it be delivered on other platforms such as through an immersive VR headset. Second, the study sample for this user experience testing study was small and potentially unbalanced. Larger sample sizes and a more rigorous study design (eg, a randomized controlled trial) should be used in future research to formally evaluate the efficacy of the VARIAT program with sufficient statistical power and without inflating the type II error rate [52,53]. Third, the present version of the VARIAT program only consisted of 2 modular domains for implicit bias training, race/SES and SOGI, with only 3 training scenarios for each module due to limitations on study resources and team expertise. Further, although these modules were developed by an interdisciplinary team of clinicians and researchers, patient communities were not involved in the design process. Future research will invite patient advisory groups into the development and refinement process of additional modules and scenarios for VARIAT to provide training in more comprehensive implicit bias domains during clinician-patient interactions. Fourth, this study used only self-reported measures developed by the study team to assess the efficacy outcomes, which may not be able to accurately measure biases that are inherently “implicit.” Future efficacy trials of the VARIAT program (and interventions alike) should incorporate validated implicit bias assessment tools such as the Implicit Association Test (IAT), which has been increasingly used by health care professionals in the existing literature [54]. Finally, several limitations of the study design should be noted. For example, this study did not restrict or record the number of times participants were allowed to undergo the training, which may have impacted usability and efficacy outcomes. Additionally, this study used an immediate pre-post training design. A more distant posttraining evaluation should be conducted to allow for examination of the impact of the modules on biases over time.

### Conclusions

This study presents a novel intervention (VARIAT) that uses immersive mobile technology to improve awareness of implicit bias related to race/SES and SOGI among Medicaid providers. This publicly available training program has found a promising avenue for future research and practice in reducing implicit bias in health care workplaces. Future research should be conducted to formally evaluate the VARIAT program with large samples and implicit bias testing measures, as well as incorporate additional training domains to provide impactful benefits to both health care professionals and their patients.

## Supplementary material

10.2196/51310 Multimedia Appendix 1 Race and socioeconomic status (SES) - module 1.

10.2196/51310 Multimedia Appendix 2 Sexual orientation gender identity (SOGI) - module 2.

10.2196/51310 Multimedia Appendix 3 User workflow.

## Abbreviations

AR: augmented reality
LGBTQ+: lesbian, gay, bisexual, transgender, queer
SES: socioeconomic status
SOGI: sexual orientation and gender identity
VARIAT: Virtual and Augmented Reality-based Implicit Association Training
VR: virtual reality
