# Supplementary material for: Smartphone-Based Virtual and Augmented Reality Implicit Association Training (VARIAT) for Reducing Implicit Biases Toward Patients Among Health Care Providers: App Development and Pilot Testing
Source: JMIR Serious Games. 2024 Mar 7;12:e51310. doi: 10.2196/51310 (PMC11004623; doi:10.2196/51310)
Supplement: Multimedia Appendix 1 [file games-v12-e51310-s001.docx]

**Multimedia Appendix 1.** Race/Socioeconomic Status (SES) - Module 1

| Case Title /Case # | Patient Info | Background | Scenario | Case Progression | Objective |
| --- | --- | --- | --- | --- | --- |
| Racial Bias-  1a | Hannah  Middle-aged, White, Female | Patient is distressed and complains of vague back pain with movement | You are the supervising physician in the ED. The resident saw Hannah and evaluated her. The Xray’s and exam do not reveal any new information. His plan is to discharge her to home with a 10 day prescription for oxycodone and recommendation for her to follow up with her orthopedist. | Resident presents patient per above.  The learner is then asked if they agree with this plan: Yes/No? | To serve as a baseline for Case 1b |
| Racial Bias-  1b | Tameeka,  18 year old, Black, Female | Patient is very emotional about her back pain, doesn’t understand the delay as she’s gotten pain meds from here multiple times before | You are the on-duty practitioner and ask her to describe the location and intensity of her pain. Tameeka informs you that she hurts a lot and begins to cry. This response is too vague to aid you in prescribing medication. You want to treat her pain but also must follow procedures and find a way to accurately assess Tameeka’s level of pain. You suggest starting with Toradol for her pain. | Patient disagrees with your plan and requests opiates instead. Her accompanying family is getting loud and frustrated. They reject hospital admission and want an opiate prescription to manage outpatient. | Scenario ends without asking learners for decision. Designed to make them uncomfortable |
| Transportation and housing instability- 2a | Lauren,  10 month old,  White, Female | Patient’s mom is talking on her phone, Lauren looks dirty but happy, another 4 year old child present who appears disheveled | Lauren has no-showed for her last 3 scheduled clinic visits and is behind on her immunizations and Well Child Checks. She has been to urgent care or the ED every month for the last 3 months for viral and ear infections. The clinic staff has tried calling many times to remind her parents that Lauren needs to be seen. Lauren’s mom arrived for today’s visit, but she is 1 hour late. There are many patients still waiting to be seen and it will be difficult to fit her in. The clinic policy is to cancel. | You are asked: Reschedule them or make an exception since she is so delayed in her vaccines? The mother does not seem to care that her daughter has had 3 appointment no-shows, has been constantly talking on her phone, and barely had time to answer basic questions. The mom says that she needs to leave in 45 minutes. | Promote leaner self-reflection on how social conditions and stigma can undermine capacity of patients to access health care and treatment |
| Transportation and housing instability- 2b | Lauren,  12 month old, White, Female | Lauren has returned for her 1 year old checkup. | Lauren is in for her 1-year-old checkup. She is 30 minutes late for her appointment. Mom appears stressed and exhausted. She still has no car and needs to rely on public transportation. | A nurse is giving you the patient history and the learner is asked: Fit Lauren in for an appointment although this would cause you to be late for your other patients or they will need to reschedule because of the clinic policy | Understand patient’s communities and how social conditions and practical logistics can undermine the capacities of patients to access health care and adhere to treatment. |
| Implicit bias and food insecurity-  3a | Participant-created avatar ‘Friend’ | Doctor (white male) is doing most of the talking- standing above ‘Friend’. Friend is sitting on the exam table, wearing a hospital gown. | ‘Friend’ has poorly controlled diabetes and hypertension. ‘Friend’ does not have the money to feed the family AND afford their medications. Need to decide what to spend their paycheck on this week. | Patient is quiet. Doctor looks like he is scolding and frustrated with the patient. Does not look the patient in the eye or pay attention to any body language cues | Understand how food insecurity and implicit bias can be experienced through physician body language and behavior. |
