# Supplementary material for: Smartphone-Based Virtual and Augmented Reality Implicit Association Training (VARIAT) for Reducing Implicit Biases Toward Patients Among Health Care Providers: App Development and Pilot Testing
Source: JMIR Serious Games. 2024 Mar 7;12:e51310. doi: 10.2196/51310 (PMC11004623; doi:10.2196/51310)
Supplement: Multimedia Appendix 2 [file games-v12-e51310-s002.docx]

**Multimedia Appendix 2.** Sexual Orientation/Gender Identity (SO/GI) - Module 2

| Case Title /Case # | Patient Info | Background | Scenario | Case Progression | Objective |
| --- | --- | --- | --- | --- | --- |
| SO/GI implicit bias-  1a | Jaiden, 2 year old, Bi-racial | You are the attending physician and observing your resident performing an admission history and physical. This is the first time you have met the patient. | Two women are present in the room, and we do not know their roles. One is white and one is black. Jaiden is bi-racial. The resident admitting the patient is visibly caught off guard about the patient having two mothers and is uncomfortable. The resident makes frequent speech errors while presenting the patient’s details. After a while, the parents seem withdrawn and do not continue to participate on rounds. | The resident continues to make speech errors, asks who the patient’s ‘real’ mom is, and makes further comments assuming the child is adopted while talking with the two mothers. Physician is asked: Did the resident make a microaggression? Should you give the resident feedback on her behavior? | Identify microaggressions and implicit bias through interpersonal communication with patients and their families. Give participants a chance to reflect on how speech can be used to commit microaggressions or enact implicit bias. |
| Waiting room and intake forms-  2a | N/A | Participant is asked to build their own waiting room and to think about items that would make waiting rooms comfortable and inclusive to the complete spectrum of SO/GI. | A nurse is calling a patient back to the exam room by his given name (Tyler). She calls multiple times and the patient does not respond because it is not her Chosen name. | Front office staff points to patient and says, “excuse me, she is calling for YOU”. Learner is asked, “Has the nurse/office staff committed a microaggression?” | Recognize and demonstrate behaviors that create a safe and welcoming environment. Identify health disparities experienced by the LGBTQ+ community, transgender, and gender non-conforming patients in receiving care |
| LGBTQ+ youth-  3a | Jack Stewart, 10 year old | Your friend is bringing her 10-year-old son, Jack, for a follow up of recent asthma exacerbation. | She mentions he has been trying on her clothes and make-up. She laughs, makes a joke and brushes it off.  Three months later, Jack comes for his annual checkup. Mom reports he has been seeming withdrawn or unhappy. She is worried he is depressed or using drugs. She also suspects he may be gay and does not know what to do. She loves him but not sure if she is ready to talk to him about it or that he is ready to talk to her. Asks for advice on what to do. | Learner is presented with options on how to proceed with this case like: Talk to Mom, Talk to Jack, or refer Jack to a counselor.  At the 3 month checkup, Mom talks about Jack’s use of her clothing and makeup and suggests he might be gay.  Participant is asked: True/False, is Jack gay? | Recognize and demonstrate the harmful behaviors associated with making assumptions on SO/GI in patient care. Provide opportunity for reflection on ways to reduce enacted implicit bias for SO/GI populations. |
