# Supplementary material for: Smartphone-Based Virtual and Augmented Reality Implicit Association Training (VARIAT) for Reducing Implicit Biases Toward Patients Among Health Care Providers: App Development and Pilot Testing
Source: JMIR Serious Games. 2024 Mar 7;12:e51310. doi: 10.2196/51310 (PMC11004623; doi:10.2196/51310)
Supplement: Multimedia Appendix 3 [file games-v12-e51310-s003.docx]

**
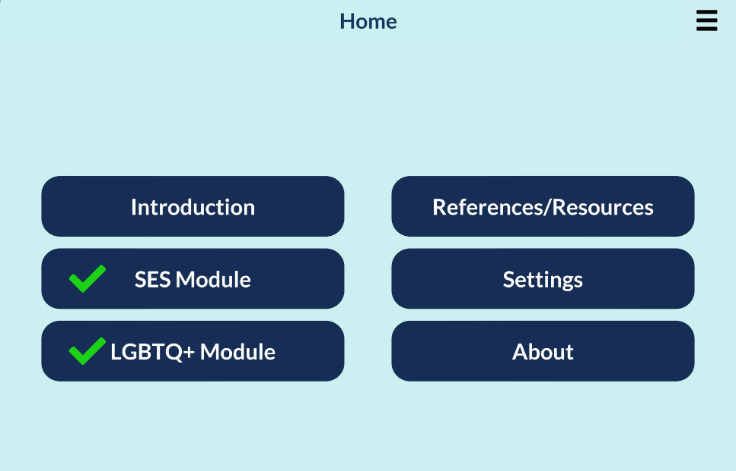
Multimedia Appendix 3.** User Workflow

**C**

**B**

**A**


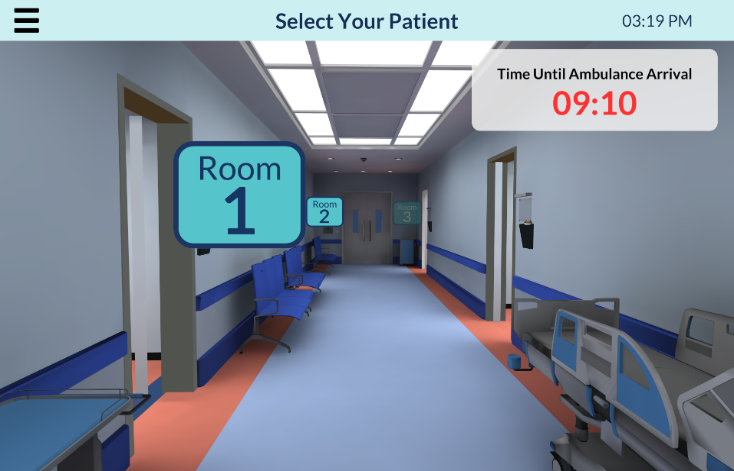

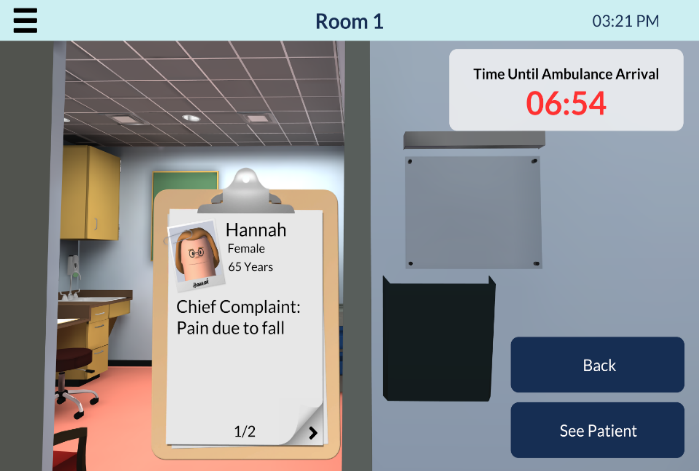


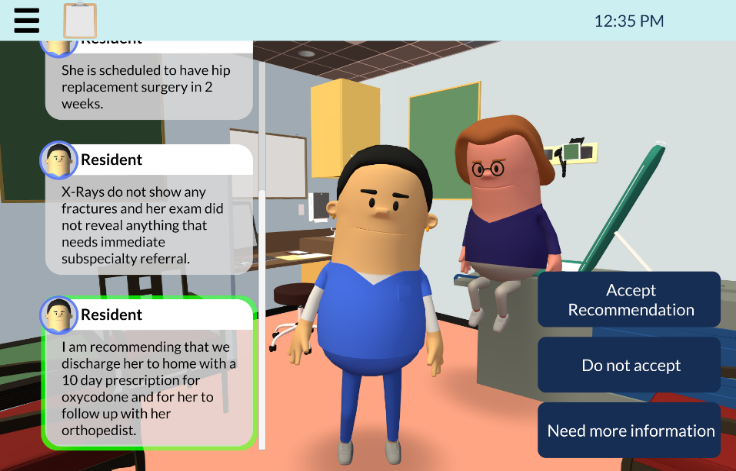

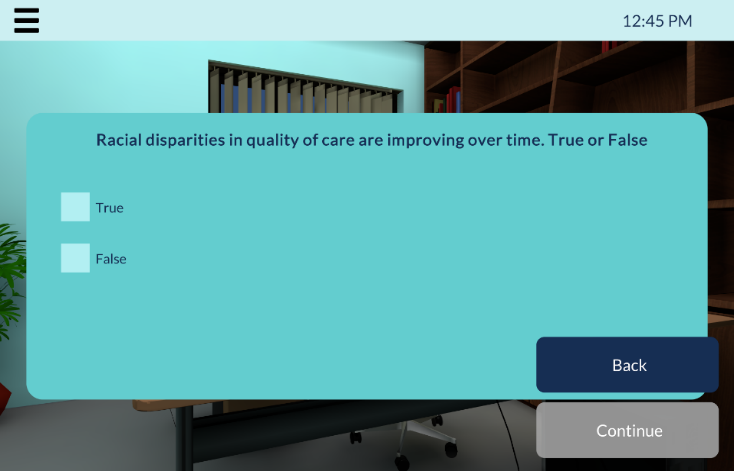

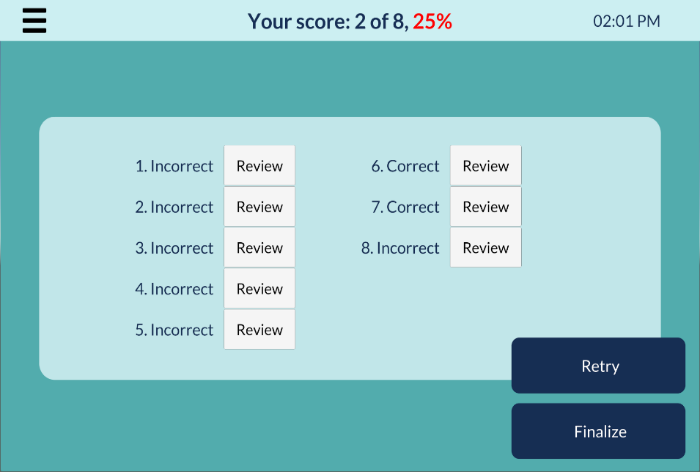


**F**

**E**

**D**

**F.** Participants are then presented with their overall scores for the exercises presented in the module and given the opportunity to review or reflect on any scenarios before retrying or choosing a different module.

**E.** Participants are then presented with questions about the overall theme of the module they selected and further prompted to reflect on the larger context of the module with information and citations about the research behind the larger issue.

**D.** Participants are then asked what decision they should make in each scenario and are given a list of options.

**C.** Selecting that room prompts participants with the patient, their chief complaint, and some information about their case.

**B.** When the module starts, participants are then immersed in a virtual hospital setting with different patients in different rooms.

**A.** Participants will open up the app on their phone/tablets and are given some information on the AR app, instructed to enter some basic demographic information, and directed to select a module to start.
